# Preoperative genetic testing impacts surgical decision making in BRCA mutation carriers with breast cancer: a retrospective cohort analysis

**DOI:** 10.1186/s13053-017-0071-z

**Published:** 2017-07-26

**Authors:** Siddhartha Yadav, Ashley Reeves, Sarah Campian, Amy Sufka, Dana Zakalik

**Affiliations:** 1 Department of Internal Medicine, Beaumont Health, 3601 W 13 Mile Rd, Royal Oak, MI 48073 USA; 2 Nancy and James Grosfeld Cancer Genetics Center, Beaumont Cancer Institute, Beaumont Health, 3577 W 13 Mile Rd, Ste. 140, Royal Oak, MI 48073 USA; 3 Oakland University William Beaumont School of Medicine, 2200 N Squirrel Rd, Rochester, MI 48309 USA

**Keywords:** BRCA, Mastectomy, Surgical decision, Surgery, Timing

## Abstract

**Background:**

The impact of timing of genetic testing on surgical decision making in women with breast cancer and *BRCA* mutation is not well known.

**Methods:**

Women who were found to carry a deleterious *BRCA* mutation and had been diagnosed with breast cancer were identified from a database at Beaumont Health. Women who had received *BRCA* positive results at least a day prior to their index surgery were considered to be aware of their mutation status prior to surgery. Baseline characteristics and surgical choices were compared between women who were aware of their mutation status prior to surgery and those who were not. Fischer’s exact test was used for categorical variables and Mann–Whitney U-Test was used for continuous variables.

**Results:**

A total of 220 patients were included in the final analysis, 208 (94.5%) with unilateral breast cancer and 12 (5.5%) with bilateral breast cancer. Out of the 208 patients with unilateral breast cancer, 106 (51.0%) patients were aware of their mutation status prior to index surgery while 102 (49%) were not. A significantly (*p* < 0.05) higher proportion of women underwent contralateral prophylactic mastectomy in the group that was aware of their mutation status prior to index surgery compared to the group that was not (76.4% vs 14.7%).

**Conclusions:**

Our study demonstrates that knowledge of *BRCA* mutation status impacts surgical decision making in favor of bilateral mastectomy in patients who are aware of their results prior to index surgery. This finding supports the practice of preoperative genetic testing in patients with newly diagnosed breast cancer.

## Background

Breast cancer is a leading cause of mortality and morbidity in women. The American Cancer Society estimates that 252,710 women will be diagnosed with invasive breast cancer and 40,610 will die from the disease in 2017 [1]. Approximately 5 to 10% of women with breast cancer carry a deleterious mutation in *BRCA1* or *BRCA2* (*BRCA1/2* hereafter) [2–4] and may be at an increased risk for recurrence of breast cancer in the same or opposite breast [5–8].

In women with *BRCA1/2* associated breast cancer, contralateral prophylactic mastectomy (CPM) markedly reduces the risk of breast cancer in the opposite breast [9–11] and may impact survival [12, 13]. Hence, in women with a newly diagnosed breast cancer, knowledge of *BRCA1/2* mutation may impact the surgical choices in favor of bilateral mastectomy. Although surgical decision making is a complex process and involves taking into consideration several factors ranging from characteristics of the tumor to personal preference, genetic testing is increasingly playing a significant role in this process in women with *BRCA1/2* mutation.

Several studies have evaluated the timing of genetic testing and impact on surgical decision making in women with breast cancer [14–19]. However, most of these studies had a small sample size of women with *BRCA1/2* mutation. In addition, the true impact of the *BRCA1/2* positive results on the extent of surgery is still not well-established and is an area of ongoing research. Hence, in this study, we evaluate the impact of timing of genetic testing on surgical decision making in a large cohort of women with *BRCA1/2* mutation.

## Methods

**Fig. 1:**
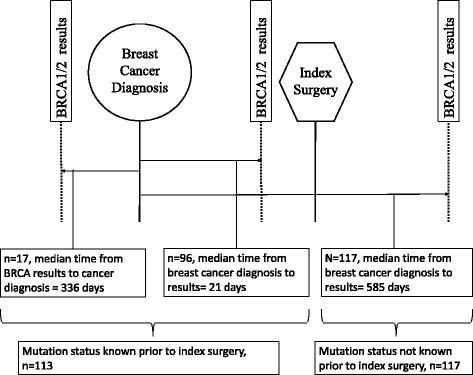
Schematic diagram demonstrating timeline of genetic testing

Women who were found to carry a deleterious *BRCA1/2* mutation were identified from patient database at Nancy and James Grosfeld Cancer Genetics Center at Beaumont Health. These patients had undergone genetic testing between January 1, 2001 and December 30, 2015. This list of women was then cross matched with cancer registry at Beaumont Health to identify women with a diagnosis of breast cancer between January 1, 1990 and December 30, 2015. These women had undergone genetic testing at different times in relation to their breast cancer diagnosis and surgery: before, during or after their diagnosis of breast cancer and/or surgery (Fig. 1). The turn-around time for genetic test results was not collected. Women with a diagnosis of breast cancer prior to or after the study timeline were excluded. Only data on surgery from the index or first surgery was collected and subsequent surgery data was not collected.

A total of 222 women met our inclusion criteria. Two women were excluded from our analysis as they had metastatic disease at diagnosis. Data on demographics, tumor characteristics and treatment was retrospectively collected from the cancer registry. Any additional data not available in the cancer registry was collected by review of electronic health records of these patients. All ‘pathogenic’ or ‘likely pathogenic’ results were considered as positive results. Women who had received the results of their genetic testing at least a day prior to their index surgery were considered to be aware of their mutation status prior to surgery. Baseline characteristics and surgical choices were compared between women who were aware of their mutation status prior to surgery and those who were not.

Data was collected in Microsoft Excel (Ver. 2007) and statistical analysis was performed using SPSS 21(IBM Corp. Released 2012. IBM SPSS Statistics for Windows, Version 21.0. Armonk, NY:IBM Corp.). Fischer’s exact test was used for categorical variables and Mann–Whitney U-Test was used for continuous variables. Bonferroni correction was used when comparing column proportions. Multivariate analysis was performed using multinomial logistic regression model. All tests were two sided. Statistical significance was considered at *p* < 0.05.

## Results

A total of 220 patients were included in the final analysis, 113 (51.4%) in the group that was aware of their mutation status prior to index surgery and 107 (48.6%) in the group that was not.

### Baseline characteristics (Table 1)

The mean age at diagnosis for all patients was 47.3 years. Majority (85.9%) of the patients were Caucasians and 12 (5.5%) had bilateral disease at presentation. The most common histology was invasive ductal carcinoma (82.3%) and the most common tumor grade was grade 3 (63.6%). Stage II was the most common (37.7%) stage at presentation. Patients who were aware of their results prior to their surgery had a younger age at diagnosis and were more likely to have received adjuvant radiation or neoadjuvant chemotherapy.

**Table 1:** Baseline characteristics

	Total (*n* = 220)	BRCA results known prior to surgery (*n* = 113)	BRCA results known after surgery (*n* = 107)	*p*-value
Age at diagnosis				
Mean age (years)	47.3	45.7	49.1	<0.05
Range (years)	22.3 – 81.7	22.3 – 79.1	26.0 – 81.7	
Median age (years)	46.4	44.0	47.6	<0.05
Race				
Caucasian	189 (85.9%)	97 (85.8%)	92 (86.0%)	NS
African American	14 (6.4%)	8 (7.1%)	6 (5.6%)	NS
Other	3 (1.4%)	2 (1.8%)	1 (0.9%)	NS
Unknown	14 (6.4%)	6 (5.3%)	8 (7.5%)	NS
Laterality				
Unilateral	208 (94.5%)	106 (93.8%)	10. (95.3%)	NS
Bilateral	12 (5.5%)	7 (6.2%)	5 (4.7%)	NS
Histology				
DCIS	24 (10.9%)	12 (10.6%)	12 (11.2%)	NS
IDC	181 (82.3%)	93 (82.3%)	88 (82.2%)	NS
ILC	13 (5.9%)	6 (5.3%)	7 (6.5%)	NS
Other	2 (0.9%)	2 (1.8%)	0 (0.0%)	NS
Grade				
Grade 1	13 (5.9%)	7 (6.2%)	6 (5.6%)	NS
Grade 2	58 (26.4%)	35 (31.0%)	23 (21.5%)	NS
Grade 3	140 (63.6%)	68 (60.2%)	72 (67.3%)	NS
Unknown	9 (4.1%)	3 (2.7%)	6 (5.6%)	NS
Tumor Size				
Mean size (mm)	27.02	30.80	22.95	<0.05
Median size (mm)	19.5	22.00	18.00	<0.05
ER status				
Negative	104 (47.3%)	51 (45.1%)	53 (49.5%)	NS
Positive	113 (51.4%)	62 (54.9%)	51 (47.7%)	NS
Unknown	3 (1.4%)	0 (0.0%)	3 (2.8%)	NS
PR status				
Negative	133 (60.5%)	67 (59.3%)	66 (61.7%)	NS
Positive	84 (38.2%)	46 (40.7%)	38 (35.5%)	NS
Unknown	3 (1.4%)	0 (0.0%)	3 (2.8%)	NS
HER-2 Status				
Negative	158 (71.8%)	89 (78.8%)	69 (64.5%)	<0.05
Positive	14 (6.4%)	11 (9.7%)	3 (2.8%)	<0.05
Not performed or unknown	48 (21.8%)	13 (11.5%)	35 (32.7%)	<0.05
Triple negative breast cancer				
Yes	80 (36.4%)	43 (38.1%)	37 (34.6%)	NS
No	92 (41.8%)	57 (50.4%)	35 (32.7%)	<0.05
Unknown	48 (21.8%)	13 (11.5%)	35 (32.7%)	<0.05
T- Stage				
Tis	24 (10.9%)	12 (10.6%)	12 (11.2%)	NS
T1	101 (45.9%)	46 (40.7%)	55 (51.4%)	NS
T2	66 (30.0%)	38 (33.6%)	28 (26.2%)	NS
T3	23 (10.5%)	12 (10.6%)	11 (10.3%)	NS
T4	6 (2.7%)	5 (4.4%)	1 (0.9%)	NS
N-Stage				
N0	133 (60.5%)	67 (59.3%)	66 (61.7%)	NS
N1	69 (31.4%)	32 (28.3%)	37 (34.6%)	NS
N2	15 (6.8%)	12 (10.6%)	3 (2.8%)	<0.05
N3	3 (1.4%)	2 (1.8%)	1 (0.9%)	NS
Overall TNM Stage				
Stage 0 (In-situ)	24 (10.9%)	12 (10.6%)	12 (11.2%)	NS
Stage I	79 (35.9%)	37 (32.7%)	42 (39.3%)	NS
Stage II	83 (37.7%)	42 (37.2%)	41 (38.3%)	NS
Stage III	34 (15.5%)	22 (19.5%)	12 (112.2%)	NS
Adjuvant radiation				
Yes	104 (47.3%)	42 (37.2%)	62 (57.9%)	<0.05
No	116 (52.7%)	71 (62.8%)	45 (42.1%)	<0.05
Chemotherapy				
None	71 (32.3%)	31 (27.4%)	40 (37.4%)	NS
Neoadjuvant chemotherapy	49 (22.3%)	43 (38.1%)	6 (5.6%)	<0.05
Adjuvant chemotherapy	100 (45.4%)	39 (34.5%)	61 (57.0%)	<0.05
BRCA1/2 results				
BRCA1 mutation	111 (50.5%)	56 (49.6%)	55 (51.4%)	NS
BRCA2 mutation	109 (49.5%)	57 (50.4%)	52 (48.6%)	NS

*NS* Not significant

### Surgical decision making in patients with unilateral breast cancer

**Fig. 2:**
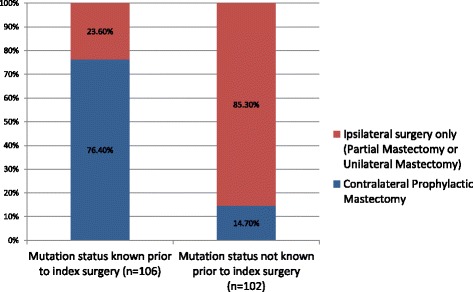
Type of index surgery in *BRCA* mutation carriers with unilateral breast cancer

Out of the 208 patients with unilateral breast cancer, 106 (51.0%) patients were aware of their mutation status prior to index surgery while 102 (49%) were not. Among those who were aware of their mutation status prior to surgery, majority (76.4%) underwent CPM during index surgery (Fig. 2). On the other hand, among patients who were not aware of their mutation status prior to surgery, a small proportion (14.7%) underwent CPM during index surgery. Majority (61.8%) of these patients underwent partial mastectomy while 24 (23.5%) underwent unilateral mastectomy (Table 2).

**Table 2:** Type of index surgery in patients with unilateral breast cancer

	Total (*n* = 208)	BRCA results known prior to surgery (*n* = 106)	BRCA results known after surgery (*n* = 102)	*p*-value
Partial mastectomy	79 (38.0%)	16 (15.1%)	63 (61.8%)	<0.05
Unilateral mastectomy	33 (15.9%)	9 (8.5%)	24 (23.5%)	<0.05
Bilateral mastectomy	96 (46.2%)	81 (76.4%)	15 (14.7%)	<0.05

### Surgical decision making in patients with bilateral breast cancer

Out of the 12 patients with bilateral breast cancer, 7 (58.3%) knew their mutation status prior to their surgery and all seven underwent bilateral mastectomy. The rest 5 (41.7%) came to know about their mutation status after their index surgery. Of these, four underwent bilateral mastectomy at index surgery while one patient underwent bilateral partial mastectomy.

### Factors associated with bilateral mastectomy

Apart from knowledge of *BRCA* mutation status prior to index surgery, other factors that were associated with a higher probability of undergoing bilateral mastectomy in univariate analysis were younger age (≤50), bilateral breast cancer, triple-negative hormone receptor status and Stage III breast cancer at diagnosis (Table 3). In multivariate analysis including all variables listed in Table 3 as covariates in addition to timing of genetics results (pre-operative vs. post-operative), bilateral breast cancer, triple negative hormone receptor status, higher overall TNM stage and pre-operative genetic testing remained significant predictors of bilateral mastectomy at index surgery.

**Table 3:** Other factors associated with bilateral mastectomy during index surgery

	Unilateral or partial mastectomy	Bilateral mastectomy	*p*-value
Age			
≤ 50 (*n* = 151)	70 (46.4%)	81 (53.6%)	<0.05
> 50 (*n* = 69)	43 (62.3%)	26 (37.7%)	<0.05
Race			
Caucasian (*n* = 189)	98 (51.9%)	91 (48.1%)	NS
African American (*n* = 14)	4 (28.6%)	10 (71.4%)	NS
Other (*n* = 3)	1 (33.3%)	2 (66.7%)	NS
Unknown (*n* = 14)	10 (71.4%)	4 (28.6%)	NS
Laterality			
Unilateral (*n* = 208)	112 (53.8%)	96 (46.2%)	<0.05
Bilateral (*n* = 12)	1 (8.3%)	11 (91.7%)	<0.05
Histology			
DCIS (*n* = 24)	13 (54.2%)	11 (45.8%)	NS
IDC (*n* = 181)	91 (50.3%)	90 (49.7%)	NS
ILC (*n* = 13)	9 (69.2%)	4 (30.8%)	NS
Other (*n* = 2)	0 (0.0%)	2 (100%)	NS
Grade			
Grade 1 (*n* = 13)	7 (53.8%)	6 (46.2%)	NS
Grade 2 (*n* = 58)	29 (50.0%)	29 (50.0%)	NS
Grade 3 (*n* = 140)	71 (50.7%)	69 (49.3%)	NS
Unknown (*n* = 9)	6 (66.7%)	3 (33.3%)	NS
Triple negative breast cancer			
Yes (*n* = 80)	34 (42.5%)	46 (57.5%)	<0.05
No (*n* = 92)	43 (46.7%)	49 (53.3%)	NS
Unknown (*n* = 48)	36 (75.0%)	12 (25.0%)	<0.05
Overall TNM Stage			
In-situ (*n* = 24)	13 (54.2%)	11 (45.8%)	NS
Stage I (*n* = 79)	50 (63.3%)	29 (36.7%)	<0.05
Stage II (*n* = 83)	39 (47.0%)	44 (53.0%)	NS
Stage III (*n* = 34)	11 (32.4%)	23 (67.6%)	<0.05

Percentages within parenthesis represent proportions within their respective rows

*NS* Not significant

### Timeline of genetic testing (Fig. 1)

Out of the 113 patient who received their results prior to surgery, 17 (15.0%) patients were aware of their mutation prior to diagnosis of breast cancer while the rest 96 (85.0%) underwent genetic testing in the interval between diagnosis and index surgery. The median time from diagnosis to test results was 21 days in the group that received their results prior to their index surgery while it was 585 days in the group that received their results after surgery.

## Discussion

Our study demonstrates that knowledge of *BRCA1/2* mutation status significantly impacts the index surgery. Majority of patients who were aware of their mutation status were able to incorporate this knowledge into surgical decision process and choose CPM. In contrast, among patients who are not aware of their mutation status at index surgery, majority choose partial mastectomy. These patients may elect to undergo a CPM at a later point to reduce their risk of contralateral breast cancer.

Our findings are consistent with several prior studies [14–19]. However, our sample size of *BRCA* positive women is the largest among all of the prior listed studies. In addition, most of the prior studies evaluated surgical decision making in patients treated at National Cancer Institute (NCI) designated comprehensive cancer centers which may not be generalizable. Our patient data originates from a community based teaching hospital. Since each year only around 250,000 patients are diagnosed with cancer at NCI designated comprehensive cancer centers [20] compared to an annual incidence of around 1.6 million new cases [1], our data may be a better reflection of actual practice in the community.

There are several important implications of our findings. Our study demonstrates that when patients and surgeons are aware of a *BRCA* mutation status prior to index surgery, they more often elect bilateral mastectomy. This preoperative genetic testing approach has several advantages including reducing the need for additional surgeries and potentially impacting survival [12, 13]. Patients who were not aware of their mutation status prior to index surgery and underwent partial mastectomy will most likely be offered bilateral mastectomy after the results of genetic testing is known [21–23], potentially incurring additional costs and morbidities.

In addition, the patients who did not know their *BRCA* status and underwent partial mastectomy will most likely receive radiation as part of breast conservation therapy, as seen in our data demonstrating the higher percentage of radiation in this group. If these patients, upon testing positive, eventually elect to undergo bilateral mastectomy, the prior adjuvant radiation may complicate reconstruction [24]. This radiation therapy, and its consequences, could have been potentially avoided in these patients. Although our study does not directly look at cost analysis, there is a potential cost-effectiveness benefit associated with genetic testing prior to surgery, as this may reduce the need for additional surgeries and radiation therapy.

Our findings along with the potential advantages discussed make a good case for changing the current practice of genetic testing in favor of preoperative genetic testing at breast cancer diagnosis. However, there are several factors that need to be aligned to obtain results of *BRCA* testing prior to index surgery. Most important of all is early identification and referral of patients who meet the guidelines for *BRCA* testing. Only around a half to two-thirds of patients who are at risk of harboring a *BRCA* mutation undergo genetic testing [25, 26]. It is unclear what percentage of patients undergoes testing prior to index surgery. A prior study suggested that if rapid testing is available and genetic referrals are made for appropriate patients, a high proportion are likely to opt for such testing [27]. Increasing the uptake of genetic testing prior to index surgery will require a multidisciplinary approach involving radiologist, pathologists and surgeons. In addition, genetic testing services with physicians and ancillary staff with expertise in genetic evaluation will have to be available to absorb any increase in uptake.

Another important factor to consider is time from ordering test to receipt of results. It is unclear whether waiting for the results of genetic testing will lead to a significant delay in surgery. The finding that there were more patients in the preoperative group who received neoadjuvant chemotherapy in our study suggests that these patients were able to obtain their results prior to surgery because of the time it took to receive the chemotherapy.

In the group that received their results prior to surgery, median time from breast cancer diagnosis to *BRCA1/2* positive results was 21 days. The concept of rapid genetic testing and counseling is evolving [28–32]. In one European study, only one third of patients who underwent rapid genetic testing and counseling were able to receive their results prior to index surgery [31]. The results might be different in the United States considering that the turnover time for results is much faster.

Although breast cancer diagnosis is known to be associated with increased levels of distress [33], the added impact of genetic counseling and testing at the time of diagnosis has not been extensively studied. This is an important additional factor to consider when recommending preoperative genetic testing. Studies are conflicting in terms of psychological distress associated with rapid genetic testing and counseling at the time of diagnosis of breast cancer, with some studies suggesting possible increased distress [28] while others suggesting no change [29, 30].

Our study also identified several factors beyond knowledge of *BRCA* status which were associated with higher rates of bilateral mastectomy (Table 3). Some of these factors such as bilateral breast cancer and higher overall stage have been known to be associated with bilateral mastectomy in previous studies [17, 34]. A prior study also found a slightly higher rate of mastectomy and bilateral mastectomy in triple negative breast cancer patients compared to estrogen receptor positive patients [35]. Our finding that patients with triple negative breast cancer more frequently underwent bilateral mastectomy needs further evaluation. This may be due to the use of neoadjuvant chemotherapy in this population allowing for more time to obtain *BRCA* testing results and may also be due to the known aggressive nature of this subtype of breast cancer.

Several limitations of our study must be pointed out. We did not collect data on family history which could also impact surgical choices. Furthermore, there was not always a temporal association between a diagnosis of breast cancer and genetic testing, as some patients for a variety of reasons had their genetic testing significantly before or after their diagnosis (Fig. 1). It is possible that some of these patients did not meet *BRCA* testing criteria or did not have access to testing at the time of their breast cancer diagnosis, which was not evaluated our study. Also, we did not control for several other factors that affect surgical decision making such as educational status, use of preoperative MRI and patient and surgeon preferences [36–39].

## Conclusions

Our study demonstrates that knowledge of *BRCA* mutation status impacts surgical decision making in favor of bilateral mastectomy in patients who are aware of their results prior to index surgery. This finding supports the practice of preoperative genetic testing in patients with newly diagnosed breast cancer and can offer potential advantages such as reduction in the need for additional surgeries and adjuvant radiation. However, further studies are needed to fully comprehend the impact of preoperative genetic testing, including assessing the feasibility of this approach on a large scale as well as delineating the psychosocial effects on patients.

## Abbreviations

BRCA1/2: BRCA1 or BRCA2
CPM: Contralateral prophylactic mastectomy
NCI: National Cancer Institute
